# Cardiometabolic prevention consultation in the Netherlands: screening uptake and detection of cardiometabolic risk factors and diseases – a pilot study

**DOI:** 10.1186/1471-2296-14-29

**Published:** 2013-02-26

**Authors:** Victor Van der Meer, Markus MJ Nielen, Anton JM Drenthen, Mieke Van Vliet, Willem JJ Assendelft, Francois G Schellevis

**Affiliations:** 1 Department of Public Health and Primary Care, Leiden University Medical Centre, Postzone V-0-P, PO Box 9600, 2300, Leiden, RC, The Netherlands; 2 NIVEL (Netherlands Institute for Health Services Research), Utrecht, The Netherlands; 3 Dutch College of General Practitioners, Utrecht, The Netherlands; 4 Van Vliet Training & Development, Utrecht, The Netherlands; 5 Department of Primary and Community Care, Radboud University Nijmegen Medical Centre, Nijmegen, The Netherlands; 6 Department of General Practice/EMGO Institute for Health and Care Research, VU University Medical Center, Amsterdam, The Netherlands

## Abstract

**Background:**

Until now, cardiometabolic risk assessment in Dutch primary health care was directed at case-finding, and structured, programmatic prevention is lacking. Therefore, the Prevention Consultation cardiometabolic risk (PC CMR), a stepwise approach to identify and manage patients with cardiometabolic risk factors, was developed. The aim of this study was 1) to evaluate uptake rates of the two steps of the PC CMR, 2) to assess the rates of newly diagnosed hypertension, hypercholesterolemia, diabetes mellitus and chronic kidney disease and 3) to explore reasons for non-participation.

**Methods:**

Sixteen general practices throughout the Netherlands were recruited to implement the PC CMR during 6 months. In eight practices eligible patients aged between 45 and 70 years without a cardiometabolic disease were actively invited by a personal letter (‘active approach’) and in eight other practices eligible patients were informed about the PC CMR only by posters and leaflets in the practice (‘passive approach’). Participating patients completed an online risk estimation (first step). Patients estimated as having a high risk according to the online risk estimation were advised to visit their general practice to complete the risk profile with blood pressure measurements and blood tests for cholesterol and glucose and to receive recommendations about risk lowering interventions (second step).

**Results:**

The online risk estimation was completed by 521 (33%) and 96 (1%) of patients in the practices with an active and passive approach, respectively. Of these patients 392 (64%) were estimated to have a high risk and were referred to the practice; 142 of 392 (36%) consulted the GP. A total of 31 (22%) newly diagnosed patients were identified. Hypertension, hypercholesterolemia, diabetes and chronic kidney disease were diagnosed in 13%, 11%, 1% and 0%, respectively. Privacy risks were the most frequently mentioned reason not to participate.

**Conclusions:**

One third of the patients responded to an active invitation to complete an online risk estimation. A passive invitation resulted in only a small number of participating patients. Two third of the participants of the online risk estimation had a high risk, but only one third of them attended the GP office. One in five visiting patients had a diagnosed cardiometabolic risk factor or disease.

## Background

This article reports data that has already been published in Dutch [1]. This has been reproduced in English, with permission from the copyright holder.

In the Netherlands (total population almost 17 million) more than 1 million people have cardiovascular disease, about 740.000 have diabetes and about 40.000 have chronic kidney disease ((pre)dialysis or transplantation) [2]. Mortality rates due to ischaemic heart disease and stroke are low compared to the rest of Europe (top-3 and top-4, respectively) [3]. Cardiovascular disease, diabetes mellitus and chronic kidney disease (further referred to as “cardiometabolic disease”) and cardiovascular mortality are highly associated with modifiable lifestyle factors such as smoking, physical inactivity and poor diet [4,5]. In the Netherlands, more than a quarter of the population currently smokes and about half of all people are overweight or obese [6,7]. These risk factors, together with biomedical indices such as glucose and cholesterol levels, blood pressure level and the family history of cardiometabolic disease [8] generate a personal risk profile which predicts the future development of cardiovascular disease and diabetes mellitus. In addition, the metabolic syndrome, with the hypertriglyceridaemic waist as its most prominent clinical criterion, is a contributing factor to global cardiometabolic risk [9].

Self-tests for glucose and cholesterol assessments and home devices for measuring blood pressure have become commercially available [10], but in the Netherlands no evidence-based cardiometabolic screening program exists within current medical practice. So far, cardiometabolic risk assessment in primary health care has been directed at case-finding, and structured, programmatic prevention is lacking.

General practitioners report to have a positive attitude towards preventing cardiometabolic disease, but they emphasize that screening should be directed at the group of patients with the highest cardiometabolic risk [11]. Therefore the Dutch College of General Practitioners, the National Association of General Practitioners and the Netherlands Society of Occupational Medicine together with three health foundations (Netherlands Heart Foundation, Dutch Diabetes Research Foundation and Dutch Kidney foundation) developed the guideline Prevention Consultation cardiometabolic risk (PC CMR) [2]. The PC CMR is based on current evidence regarding cardiometabolic risk estimation and comprises of a stepwise approach. Based on an online risk estimation (first step), high risk patients are referred to the general practice (second step), where the risk profile is completed and appropriate interventions are initiated. The prototype of the PC CMR was implemented in 16 general practices throughout the Netherlands for a period of 6 months. Aims of the study were 1) to evaluate uptake rates of the two steps of the PC CMR; 2) to assess the rates of newly diagnosed patients with hypertension, hypercholesterolemia, diabetes and chronic kidney disease at risk for cardiometabolic disease; and 3) to explore reasons for non participation.

## Methods

### Patients

Sixteen general practices (49 general practitioners (GPs) and 27 practice nurses) were recruited who were willing to implement the prototype of the PC CMR. Eligible patients were aged between 45 and 70 years and had no cardiovascular disease, diabetes mellitus and/or chronic kidney disease according to their electronic patient record (Table 1). In the Netherlands, GPs have a fixed practice list, and all non-institutionalized inhabitants are obligatory listed in a general practice.

**Table 1:** **Exclusion criteria for prevention consultation cardiometabolic risk (ICPC-codes [International classification of primary care]) *(not reported previously)***

**ICPC code**	**Title**
K74	Ischaemic heart disease with angina pectoris
K75	Acute myocardial infarction
K76	Ischaemic heart diseases without angina pectoris
K77	Heart failure
K78	Atrial fibrillation
K79	Paroxysmal tachycardia
K82	Pulmonary heart disease
K83	Heart valve disease
K84	Other disease of heart
K86	Uncomplicated hypertension
K87	Hypertension with involvement target organs
K89	Transient cerebral ischemia
K90	Stroke/cerebrovascular accident
K91	Atherosclerosis
K92	Peripheral vascular diseases
T90	Diabetes mellitus
T93	Lipid metabolism disorder
U88	Glomerulonephritis/nephrosis
U99	Other disease urinary system

Eight practices identified all eligible patients born in 1939, 1946, 1952, 1958 or 1964, sent them a personal letter and invited them to complete the online risk estimation (‘active approach’). This selection procedure was based on practical reasons regarding implementation. By choosing 5 birth cohorts spread out over the actual age of 45 – 70 years, we were able to reach a wide age range and participating GPs had a practical and uniform tool to invite their patients. It was not feasible for participating GPs to invite all persons between 45–70 years, since all GPs had their regular GP practice duties and activities. The first invitation letter was sent between October 2009 and January 2010. A reminder letter was sent in March 2010.

The eight other practices passively invited all eligible patients by a poster in the waiting room of the practice and by leaflets in the waiting room and consulting room (‘passive approach’). The poster and leaflets contained information on the purpose of the PC CMR and invited patients to complete the online risk estimation. Poster and leaflets were present between October 2009 and May 2010.

A medical ethics committee approval was not required according to Dutch legislation.

### PC CMR

The first step of the PC CMR is an online risk estimation. The online risk estimation was offered in a non-secure, open web environment.

**Figure 1:**
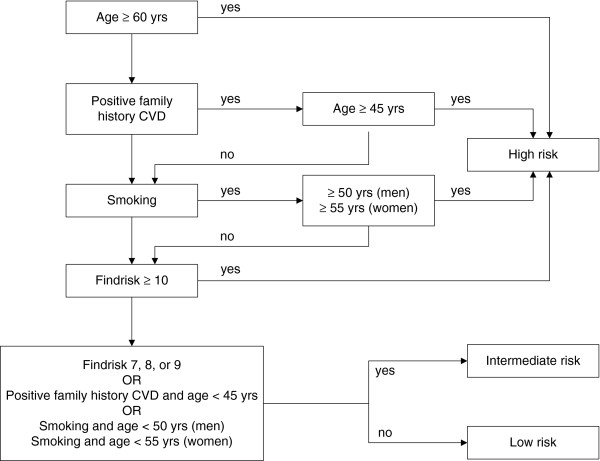
**Algorithm for estimating cardiometabolic risk *(not published previously).***

The online risk estimation consists of a web-based questionnaire on risk factors for cardiovascular disease, diabetes mellitus and chronic kidney disease [12]. The questionnaire contains three items of the Systematic Coronary Risk Evaluation (SCORE) risk function: age, gender and smoking status [13] and all items of the Finnish Diabetes Risk Score (FINDRISK): height, weight, waist circumference, history of high blood glucose and family history of diabetes mellitus [14]. Body mass index (weight (kg)/height^2^ (m^2^)) was derived from the web-based questionnaire. Since a positive family history doubles the future risk, a question on family history of cardiovascular disease was added [8]. A positive family history of cardiovascular disease was defined as a first degree relative with cardiovascular disease below the age of 65 years. Based on the algorithm in Figure 1 participants were categorized as having a low, intermediate or high risk for cardiometabolic disease.

In the second step of the PC CMR, patients estimated as having high risk according to the online risk estimation are advised to visit their general practice in order to complete their risk profile and discuss follow-up treatment. The risk profile includes assessments of serum cholesterol ratio (total cholesterol: HDL), serum glucose level and blood pressure measurements.

### Measurements

#### Uptake and participation

A representative of each general practice (either a general practitioner or practice nurse) identified the eligible population (patients between 45 and 70 years without cardiometabolic disease) by using the electronic medical records. An anonymised list of the eligible population was sent to the researchers.

Results of the online risk estimations, completed by the participants, were saved in a web-based log file. From the log file, which contained all answers to the questions of the online risk estimation, we were able to calculate the uptake of the first step of the PC CMR and to categorize the participants as having low, intermediate or high risk. Response rates to the second step of the PC CMR were calculated on the basis of the GP’s insurance claims of practice visits.

#### Cardiometabolic disease

After the study period of 6 months, GPs provided data from the electronic medical records of patients who consulted the practice on the basis of the estimated high risk at the online risk estimation. GPs reported the presence or absence of hypertension, hypercholesterolemia, diabetes mellitus and chronic kidney disease. Additionally, they reported results of diagnostic assessments and laboratory tests on blood pressure, serum cholesterol, LDL and HDL levels, serum glucose, glycated hemoglobin, serum creatinine, creatinine clearance according to the Modification of Diet in Renal Disease (MDRD) formula [15] and urine albumin-to-creatinine ratio. Finally, new prescriptions for antihypertensive medication, statins and oral antidiabetics were reported.

#### Reasons for non-response

We conducted a survey among the eligible population in order to evaluate differences between responders and non-responders. A questionnaire was sent to all eligible patients in the practices that had used the ‘active approach’, and to a random sample of 200 persons of the eligible population in practices that had invited patients passively. The questionnaire contained items on demography, health risk behaviour and attitudes towards the PC CMR. Alcohol use of >6 drinks/day was used to describe the proportion of participants with excessive alcohol abuse [16].

### Statistical analysis

We evaluated uptake rates and the incidence of cardiometabolic disease as a percentage of the eligible population. Additionally, we reported the number needed to screen (NNS) as the inverse of the proportion of patients diagnosed with a cardiometabolic disease.

We calculated statistical differences between participants and non-participants by two sample Student *t*-tests for continuous outcomes and Chi-square tests for dichotomous outcomes. We used the statistical software package STATA 10.0 (StataCorp; College Station TX, US).

## Results

All results have been previously reported in Dutch [1], except for Tables 2 and 3 and Figure 1.

**Table 2:** **Results from the online risk estimation; means and percentages *(not reported previously)***

	**Approach**		**P value**
	**Active (n = 521)**	**Passive (n = 96)**	
Age, years, mean	54,5	55,7	0,17
BMI, kg/m^2^, mean	25.4	26.8	<0,01
Gender, n (%)			
Male	216 (41,5%)	41 (42,7%)	0,82
Female	305 (58,5%)	55 (57,3%)	
Waist circumference man, n (%)			
< 94 cm	50 (23,2%)	12 (29,3%)	0,33
94 – 102 cm	101 (46,8%)	14 (34,2%)	
> 102 cm	65 (30,1%)	15 (36,6%)	
Waist circumference woman, n (%)			
< 80 cm (%)	42 (13,8%)	8 (14,6%)	0,96
80 – 88 cm (%)	106 (34,8%)	18 (32,7%)	
> 88 cm (%)	157 (51,5%)	29 (52,7%)	
Ever high blood glucose or diabetes, n (%)			
Yes	33 (6,3%)	3 (3,1%)	0,22
No	488 (93,7%)	93 (96,9%)	
Family history of diabetes, n (%)			
Yes	143 (27,5%)	30 (31,3%)	0,45
No	378 (72,6%)	66 (68,8%)	
Family history of cardiovascular disease, n (%)			
Yes	134 (25,7%)	32 (33,3%)	0,12
No	387 (74,3%)	64 (66,7%)	
Smoking, n (%)			
Yes	109 (20,9%)	20 (20,8%)	0,98
No	412 (79,1%)	76 (79,2%)	

**Table 3:** **Diagnostic test results, diagnoses and prescribed medication of 142 GP office visitors; percentages, and calculated number to screen among high risk patients *(not reported previously)***

	***Based on N***	***Identified N (%)***	NNS*
Blood pressure			
Hypertension	142	18 (12.7%)	7.9
Systolic blood pressure ≥180 mmHg	131	5 (3.8%)	26.2
Cholesterol			
Hypercholesterolemia	142	15 (10.6%)	9.5
Cholesterol ≥ 8.0 mmol/l or cholesterol/HDL-ratio ≥ 8.0	126	5 (4.0%)	25.5
Glucose			
Diabetes mellitus	142	2 (1.4%)	70.9
Impaired Fasting Glucose (≥6.1 and ≤6.9 mmol/l)	120	14 (11.7%)	8.6
Kidney function			
Chronic kidney disease	142	0 (0%)	∞
Urine albumin-to-creatinine ratio >3.5 mg/mmol	49	2 (4.1%)	24.5
Medication			
Antihypertensive agents	142	11 (7.8%)	12.9
Statins	142	5 (3.5%)	28.4
Blood glucose lowering drugs	142	3 (2.1%)	47.4

* NNS: Number needed to screen.

### Uptake and cardiometabolic disease

**Figure 2:**
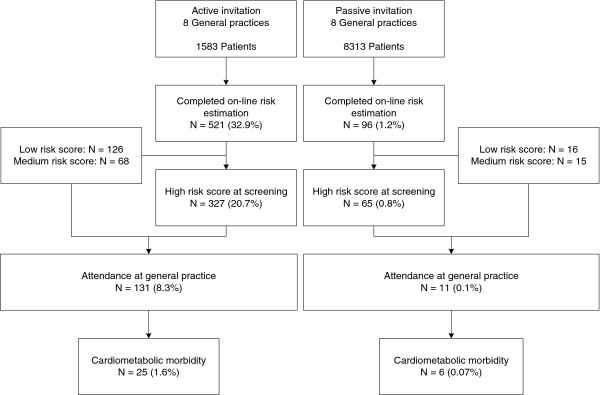
Flow chart of the results of the prevention consultation cardiometabolic risk.

In the 8 practices using the active approach, 1,583 patients received an invitation letter to participate in the PC CMR. Of these, 521 (32.9%) completed the online risk estimation (Figure 2). Their mean age was 54 years and 59% were women. In 283 (54.3%) cases the online questionnaire was completed after the date a reminder letter was sent.

The eligible population in the 8 practices using the passive approach consisted of 8,313 patients. Ninety-six (1.2%) patients completed the online risk estimation (Figure 2). Their mean age was 56 years and 57% were women. Their age and gender did not statistically significantly differ from participants in the active approach (p = 0.17 and p = 0.82, respectively).

Table 2 shows the results of the online risk estimation. Participants from practices with the passive approach had a higher body mass index than patients from practices in the active approach (25.4 versus 26.8, p < 0.01). Of all participants, 129 (21%) smoked, 253 (41%) were overweight (BMI ≥ 25 and <30), 74 (12%) were obese, and 36 (6%) had a history of high blood glucose or diabetes mellitus. A positive family history of diabetes mellitus and cardiovascular disease was reported by 173 (28%) and 166 (27%), respectively.

A total of 392 (63.5%) participants had a high risk for cardiometabolic disease, based on the online risk estimation; 83 (13.5%) and 142 (23.0%) participants had an intermediate or low risk, respectively. There was no statistically significant difference of the distribution of estimated risk profiles between the participants in the two types of practices.

Only patients with a high risk score were advised to visit the practice. A total of 142 participants visited the practice. Nine percent of the visitors had a low or intermediate score at the online risk estimation, but nevertheless visited the GP office despite the negative advice. Results of diagnostic tests, and prescribed medication are shown in Table 3. Eighteen participants (13%) had a newly diagnosed hypertension, 15 (11%) hypercholesterolemia, two (1%) had diabetes mellitus and two (4%) had albuminuria. Four participants (3%) had both hypertension and hypercholesterolemia. Based on a physician’s diagnosis of hypertension, hypercholesterolemia, diabetes mellitus or chronic kidney disease, a total of 31 newly diagnosed patients (22%) were identified.

### Non-response

The questionnaire to assess the reasons for non-response was sent to 3,183 patients of whom 932 (29.3%) returned the questionnaire (427 from practices with the active approach, and 505 from practices with the passive approach). A large proportion of patients listed in practices of the passive approach answered that they were not familiar with the PC CMR (n = 433 (85.7%)). Of these 433 patients, 203 (46.9%) had not visited the practice in the past six months and have therefore not been able to take notice of a poster of leaflet in the practice about the PC CMR.

For the analysis of reasons for non-response data were available of 274 patients (29% of the questionnaire responders) who reported that they only had completed the first step of the PC CMR (the online risk estimation) and 177 patients who were familiar with the PC CMR (had seen or heard about it), but had not completed the online risk estimation.

Age, gender, marital status, education level and ethnic background did not differ between responders and non-responders (Table 4). There were only small, non-significant differences between responders and non-responders with regard to smoking status, physical activity and body mass index. Non-responders more often excessively used alcohol than responders (p < 0.001) and less often had a history of high blood sugar (p = 0.03).

**Table 4:** Demographic characteristics and health risk behaviour of responders and non-responders to online risk estimation

	**Completed online risk estimation**		**P value**
	**Yes (n = 274)**	**No (n = 177)**	
***Age***			
40-45 yr	27.3%	29.6%	0.83
46-50 yr	13.1%	12.5%	
51-55 yr	24.8%	27.3%	
56-60 yr	22.5%	19.3%	
61-65 yr	18.5%	15.3%	
66-70 yr	2.9%	4.0%	
> 70 yr	4.0%	4.5%	
***Gender***			
Male	32.5%	39.2%	0.15
Female	67.5%	60.8%	
***Marital status***			
Single	14.6%	22.2%	0.12
Partner, not living together	3.3%	2.8%	
Married and/or living together	82.1%	75.0%	
***Level of education***			
Low	22.8%	28.9%	0.33
Middle	50.0%	47.4%	
High	27.2%	23.7%	
***Ethnicity***			
Dutch	83.1%	87.5%	0.37
Western immigrant	9.9%	6.25%	
Non-western immigrant	7.0%	6.25%	
***Smoking***			
Current smoker	16.8%	22.6%	0.18
Former smoker	43.1%	35.6%	
Never smoker	40.1%	41.8%	
***Physical activity recommendation***			
Inactive lifestyle	3.7%	5.2%	0.69
Moderately active lifestyle	53.1%	54.1%	
Active lifestyle	43.2%	40.7%	
***Body mass index (BMI)***			
Underweight	4.8%	7.5%	0.11
Optimal weight	45.8%	40.5%	
Overweight	40.2%	36.4%	
Obesity	9.2%	15.6%	
***Alcohol use >6 units/day***			
Never	65.6%	49.4%	<0.001
Seldom	21.6%	21.6%	
Sometimes	9.9%	21.6%	
Often	2.9%	7.4%	
***History of high blood glucose or diabetes***	8.9%	3.5%	0.03
***Family history of diabetes***	31.4%	24.6%	0.12
***Family history of cardiovascular disease***	33.7%	29.0%	0.29

**Figure 3:**
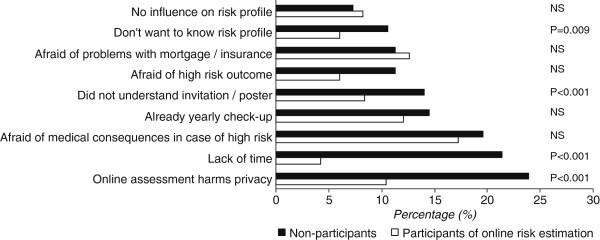
Attitude towards participating in online risk estimation of PC CMR.

The most frequently mentioned reason for not participating was the fear that online assessment is a privacy risk (23.9%) (Figure 3). Other frequently mentioned reasons were lack of time (21.4%) and fear of medical consequences related to high-risk assessment (19.6%). It must be noted that patients who did not participate more often reported difficulties accessing the internet than participants (24.1% vs 10.9%, p < 0.001).

## Discussion

We evaluated uptake rates, newly detected cardiometabolic disease and reasons for non-response of the newly developed Prevention Consultation cardiometabolic risk (PC CMR) in a 6-month, multi-center implementation study. The uptake rates of both steps of the PC CMR were substantially higher in practices that actively invited patients to participate compared to practices that only used leaflets and posters to invite patients. In one out of five patients who attended the GP office a cardiometabolic disease, defined as hypertension, hypercholesterolemia, diabetes or chronic kidney disease was diagnosed. Limited access to the internet and the fear that participation in the PC CMR is a privacy risk were the major reasons for non-participation.

### Participation

Although patients who were actively invited to participate in the health check more often participated than patients who were passively invited, only one third of the eligible group completed the online risk estimation. In the Netherlands 87% of all inhabitants have home access to internet [17], ranging from 79% in low educated to 95% in high educated persons. Apparently, this high internet coverage does not guarantee a high participation in an online risk estimation. Non-participants mention concerns regarding privacy with online assessments as the most important reason not to participate. Other studies show that paper-and-pencil questionnaires, sent to an eligible population, results in participation rates up to 75% [18,19]. In our study the PC CMR was offered in a non-secure, open web environment. Participants were able to complete the online questionnaire without the use of a log-in account or SMS authentication. It remains questionable whether the use of a more secured web environment or a better explanation about the privacy within the project would increase participation.

Previous studies suggest that participants of preventive health checks are better educated, better motivated to look after their health and perform more health-approved practices than non-participants [18,20,21]. However, our evaluation shows that participation was not confined to the worried-well: the prevalence of smoking, physical inactivity and overweight did not differ between responders and non-responders. It must be noted that the number of non-Western immigrants in the analyses was low, which is remarkable since four participating practices were located in the multi-cultural city of Rotterdam. We recommend that with further implementation of the Prevention Consultation in the Netherlands paper questionnaires are used beside online risk assessments and that both paper and online questionnaires are available in different languages.

### Detection of cardiometabolic risk factors and disease

This study identified large numbers of smokers, patients with overweight and/or physical inactivity. Recent Dutch guidelines (on smoking and on obesity) for primary care emphasize the need to guide and treat patients with these modifiable cardiovascular risk factors [7,22]. The easily accessible and integral setting of primary care is the ideal place to guide these patients to a healthier lifestyle. Moreover, our study identified one quarter of participants with a positive family history of cardiovascular disease or diabetes. Although a validated treatment algorithm is lacking, intensified follow-up or risk management is justifiable for this group at relatively high risk of cardiovascular disease [8].

In 22% of patients with a high risk who attended the practice a cardiometabolic disease (hypertension, hypercholesterolemia, diabetes) was diagnosed. Obviously, these patients may benefit from lifestyle advice and cardiovascular follow-up assessments. Whether this group also needs drug treatment depends on the integrated cardiometabolic profile.

### Limitations

Three issues regarding the used outcome measures need attention. First, the diagnoses hypertension and hypercholesterolemia were based on physician’s records and not on absolute cut-off points for blood pressure or cholesterol. In fact, absolute cut-off points do not exist (anymore), since cardiovascular risk management depends on the integrated profile and not on a single blood pressure (SBP) or cholesterol [13]. For example, according to the Dutch guidelines, a 55-year old man who smokes, has an SBP of 160 and cholesterol ratio of 8 needs drug treatment, whereas a 55-year old non-smoking man with a similar SBP of 160, but cholesterol ratio of 4 may not need drug treatment. Although a physician’s diagnosis of hypertension or hypercholesterolemia probably differs between professionals, our approach reflects current daily medical practice. Second, the outcome measures of this implementation study are limited by the fact that measurements were only taken once (blood pressure, blood and urine tests). Follow-up measurements are warranted to establish a more valid diagnosis. Third, obviously the presented outcomes are intermediate measures. The design and time frame of the study did not allow analysis of endpoints such as cardiovascular morbidity and morbidity.

## Conclusion

Nationwide implementation of the Prevention Consultation in general practice is likely to be successful when patients are approached actively (with a reminder) and are able to complete the cardiometabolic risk estimation not only online, but also using a written questionnaire in multiple languages. The privacy of all assessments needs to be guaranteed and this should be made clear to eligible participants.

The Prevention Consultation seems to adequately detect cardiometabolic risk factors and diseases in those patients who attend the practice. Future efforts for both professionals and researchers should be directed towards longitudinal follow-up, lifestyle coaching and drug treatment in order to assess the effects of the Prevention Consultation cardiometabolic risk on long-term morbidity and mortality.

## Abbreviations

GP: General Practitioner; MDRD: Modification of Diet in Renal Disease; NNS: Number Needed to Screen; PC CMR: Prevention Consultation cardiometabolic risk; SBP: Systolic blood pressure.

## Competing interests

The authors declare that they have no competing interests.

## Authors’ contributions

VvdM, MN, WA and FS contributed to conception and design. VvdM and MN acquired and analysed the data. All authors interpreted the data and were involved in drafting and revising the manuscript. All authors read and approved the final manuscript.

## 

This is an elaborated and translated version of a recently published article in ‘Huisarts en Wetenschap’ (in Dutch).

## Funding

This study was supported by a grant from the governmental Netherlands Organization for Health Research and Development (ZonMw grantnr 50-50115-96-700).

## Pre-publication history

The pre-publication history for this paper can be accessed here:

http://www.biomedcentral.com/1471-2296/14/29/prepub
